# Predicting Survival in Patients with Brain Tumors: Current State-of-the-Art of AI Methods Applied to MRI

**DOI:** 10.3390/diagnostics12092125

**Published:** 2022-09-01

**Authors:** Christian di Noia, James T. Grist, Frank Riemer, Maria Lyasheva, Miriana Fabozzi, Mauro Castelli, Raffaele Lodi, Caterina Tonon, Leonardo Rundo, Fulvio Zaccagna

**Affiliations:** 1 Department of Biomedical and Neuromotor Sciences, Alma Mater Studiorum—University of Bologna, 40125 Bologna, Italy; 2 Department of Physiology, Anatomy, and Genetics, University of Oxford, Oxford OX1 3PT, UK; 3 Department of Radiology, Oxford University Hospitals NHS Foundation Trust, Oxford OX3 9DU, UK; 4 Oxford Centre for Clinical Magnetic Research Imaging, University of Oxford, Oxford OX3 9DU, UK; 5 Institute of Cancer and Genomic Sciences, University of Birmingham, Birmingham B15 2SY, UK; 6 Mohn Medical Imaging and Visualization Centre (MMIV), Department of Radiology, Haukeland University Hospital, N-5021 Bergen, Norway; 7 Division of Cardiovascular Medicine, Radcliffe Department of Medicine, University of Oxford, John Radcliffe Hospital, Oxford OX3 9DU, UK; 8 Centro Medico Polispecialistico (CMO), 80058 Torre Annunziata, Italy; 9 NOVA Information Management School (NOVA IMS), Universidade NOVA de Lisboa, Campus de Campolide, 1070-312 Lisboa, Portugal; 10 Functional and Molecular Neuroimaging Unit, IRCCS Istituto delle Scienze Neurologiche di Bologna, 40139 Bologna, Italy; 11 Department of Information and Electrical Engineering and Applied Mathematics, University of Salerno, 84084 Fisciano, Italy

**Keywords:** brain tumors, artificial intelligence, machine learning, survival prediction, magnetic resonance imaging

## Abstract

Given growing clinical needs, in recent years Artificial Intelligence (AI) techniques have increasingly been used to define the best approaches for survival assessment and prediction in patients with brain tumors. Advances in computational resources, and the collection of (mainly) public databases, have promoted this rapid development. This narrative review of the current state-of-the-art aimed to survey current applications of AI in predicting survival in patients with brain tumors, with a focus on Magnetic Resonance Imaging (MRI). An extensive search was performed on PubMed and Google Scholar using a Boolean research query based on MeSH terms and restricting the search to the period between 2012 and 2022. Fifty studies were selected, mainly based on Machine Learning (ML), Deep Learning (DL), radiomics-based methods, and methods that exploit traditional imaging techniques for survival assessment. In addition, we focused on two distinct tasks related to survival assessment: the first on the classification of subjects into survival classes (short and long-term or eventually short, mid and long-term) to stratify patients in distinct groups. The second focused on quantification, in days or months, of the individual survival interval. Our survey showed excellent state-of-the-art methods for the first, with accuracy up to ∼98%. The latter task appears to be the most challenging, but state-of-the-art techniques showed promising results, albeit with limitations, with C-Index up to ∼0.91. In conclusion, according to the specific task, the available computational methods perform differently, and the choice of the best one to use is non-univocal and dependent on many aspects. Unequivocally, the use of features derived from quantitative imaging has been shown to be advantageous for AI applications, including survival prediction. This evidence from the literature motivates further research in the field of AI-powered methods for survival prediction in patients with brain tumors, in particular, using the wealth of information provided by quantitative MRI techniques.

## 1. Introduction

Artificial intelligence (AI) is a branch of computer science that has been successfully applied to the analysis and extraction of meaningful features from medical images, with various clinical applications [1]. In particular, the use of AI in brain imaging has been fruitful, showing promising results and generating new perspectives for diagnosis, prognosis and treatment planning [2,3,4,5].

Brain tumors are among the top ten causes of death from cancer [6,7] and can be metastatic or primary. Gliomas account for about 80% of primary malignant brain tumors; they include different sub-types of which the most common are glioblastoma (GBM), astrocytoma, oligodendroglioma, and ependymoma [8,9]. Some subtype-specific characteristics, such as cell invasion and proliferation, angiogenesis, apoptosis, and the high degree of heterogeneity contribute to both increased morbidity and mortality [10]. Among gliomas, GBM is the most aggressive and heterogeneous (at tissue, cellular and molecular level), with the highest short-term mortality rate [11,12]. Currently, the average 5-year survival rate for GBM is 5.6–7%, while the median survival is about 12–15 months [13,14,15,16]. Despite aggressive management with surgery, radiotherapy, and chemotherapy, overall patient survival (OS) remains dismal [15,17,18,19]. Magnetic Resonance Imaging (MRI) is the modality of choice in neuro-oncology for diagnosis, treatment response evaluation and prognosis prediction; since it is non-invasive and can convey a considerable amount of information about both the tumor and the surrounding areas [20,21]. Several MR techniques are routinely used to image brain tumors, aiding management from diagnosis to therapy assessment [22,23,24], and many novel techniques are in active development [20,25], however current imaging is insufficient.

Partially prompted by this unmet need, recent years have seen an increasing interest in applying AI techniques to MRI. Great emphasis has been placed on radiomics, a technique which aims to extract quantitative and reproducible features from images, including complex patterns that are often not visible to the human eye [26,27]. Specifically, radiomics refers to high-throughput extraction of quantitative features, that result in the conversion of images into mineable data, and the subsequent analysis of these data for decision support [28]. This technique has been applied to several imaging modalities including Ultrasound (US) [29], Computed Tomography (CT) [30] and MRI [31]. These approaches have been primarily applied to oncology, although there has been a growing interest also in cardiovascular applications [30,32,33,34]. Through the study of quantitative features extracted from MR images, by computing local macro- and micro-scale morphological changes in texture patterns, radiomics can accurately reflect the underlying pathophysiology of the disease by capturing statistical inter-relationships between voxels under examination [6,11,35,36,37,38,39].

A growing body of evidence suggests that radiomic analysis of MR images, can aid OS prediction, while also influencing patient management [40,41]. Therefore, those “surrogate” predictors of patient survival are of fundamental clinical interest: in particular, prediction of OS and survival classification (SC) in groups (long-term and short-term survival—survival stratification), as they would be of utmost importance in treatment evaluation, and follow-up management [40,41,42,43]. Different methods based on Machine Learning (ML) and Deep Learning (DL), and related algorithms, have been proposed to address this need for assessing survival. Traditional ML-based methods, such as support vector machines (SVMs), k-nearest neighbors algorithm (k-NN), and random forests (RFs) are generally utilized for brain tumor survival analysis. However, these ML-based methods have the common limitation of hand-crafted feature extraction [44]. DL-based methods overcome the drawback of hand-crafted feature extraction [45,46], having the ability to learn and self-determine the best features to use in a prediction model [47]. These DL methods, based on completely different approaches, have shown different performance in SC and prediction of OS. Despite the advantages and disadvantages of both ML and DL methods, establishing which of one is better is not possible, since performance of the various algorithms may vary depending on the specific task (OS or SC) and on the composition and quality of the dataset. The focus of this narrative review is to explore currently-published AI techniques applied to MRI for OS prediction and long/short term SC in patients with brain tumors. Several methods that have been proposed over the last 10 years, have been investigated. In the results section, ML and DL algorithms are presented in order of their performance.

This manuscript is organized as follows. Section 2 describes the methodology adopted in the literature review. The selected papers are briefly described in Section 3. Discussions and final remarks are presented in Section 4.

## 2. Methods

### 2.1. Literature Review

**Figure 1:**
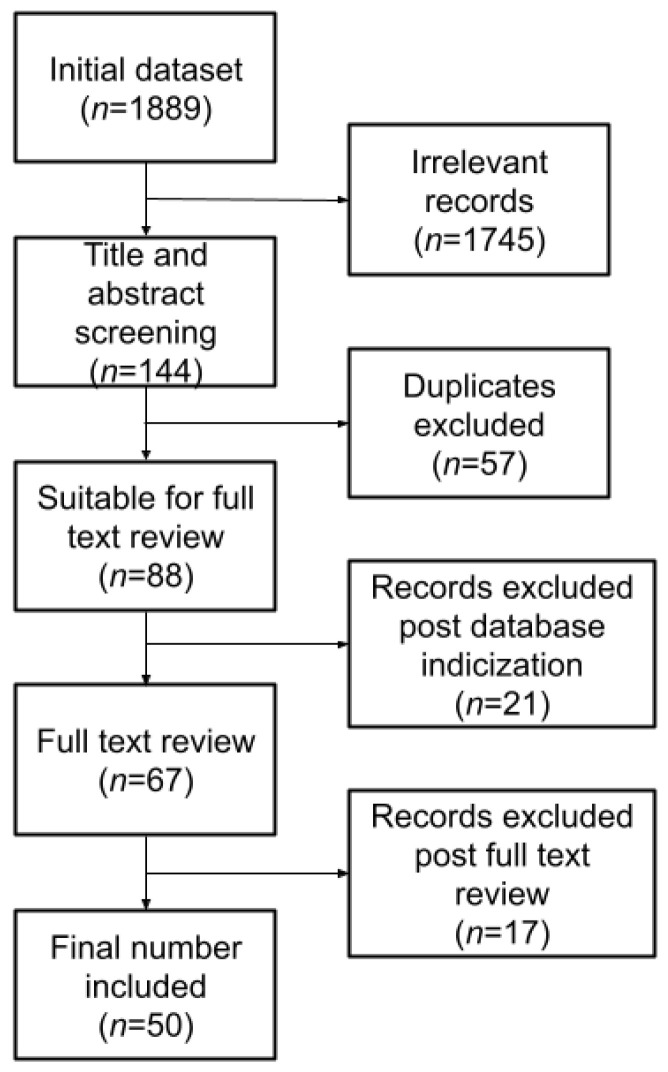
PRISMA diagram: flowchart showing screening and selection of relevant papers. For a description of the selection process, please refer to Section 2.1.

A literature review was performed according to the Preferred Reporting Items for Systematic Reviews and Meta-Analyses (PRISMA) guidelines (Figure 1). PubMed and Google Scholar databases were searched to identify all potentially relevant studies back to 1 January 2012. The search query was built using medical subject headings (MeSH) related to AI and brain. The following search query was used on both databases, restricted to original articles published between 2012–2022:

(“Machine Learning” OR “artificial intelligence” OR “Deep Learning”) AND brain AND (tumor OR tumour) AND (survival OR “life expectancy”) AND (pediatric OR paediatric OR adults) AND (MRI OR “magnetic resonance”)

All studies evaluating AI and ML models for survival prediction in patients with brain tumors were included in this study. The initial search returned 1889 results (59 from PubMed, 1830 from Google Scholar), with a significant imbalance of results from Google Scholar. Following manual elimination of duplicates, titles were carefully screened to identify relevant papers. Any work that matched at least one of the following exclusion criteria was excluded:

- no full-text available;
- no AI application or non-pertinent application;
- conference proceedings;
- books or book chapters;
- non-English manuscripts.

Review of the titles narrowed the results to 144 articles, 59 papers from PubMed and 85 from Google Scholar. Subsequently, review of the abstracts further narrowed the results to 88 articles, 35 papers from PubMed and 53 from Google Scholar. Despite review of titles and abstracts, not all the articles found on Google Scholar were relevant, moreover, some were not indexed in PubMed, and, considering the target audience and the push towards translational applications, we decided to include in this survey only indexed papers. Hence, after final revision, 50 papers (24 from PubMed, 26 from Google Scholar) were deemed eligible and included in this review.

### 2.2. Metrics

Several metrics can be used to evaluate a model, the most popular and well known are accuracy, sensitivity, specificity, and the area under the receiver operating characteristic curve (AUC), illustrating the diagnostic ability of a binary classifier system as its cut-off value varies. When estimating the goodness of a model that predicts survival, using the concordance index (C-Index) as an assessment metric may be useful. To account for the heterogeneity of the methods among the selected papers, C-Index and accuracy were used as comparison metrics for the SC and OS tasks respectively.

## 3. Results

**Figure 2:**
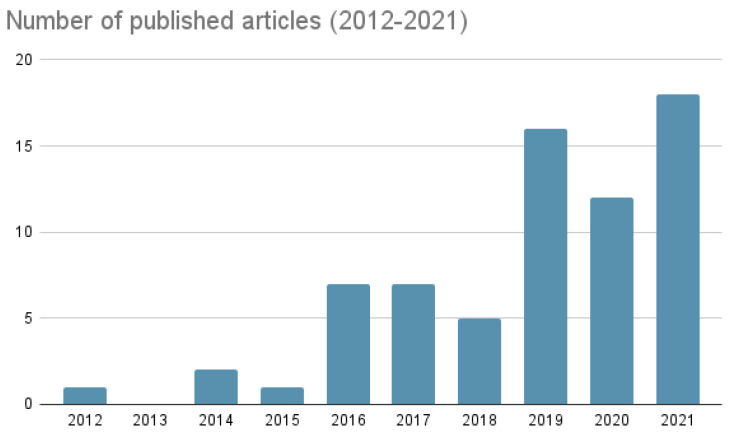
Graphical representation of the number of articles included in this review: This bar plot depicts the papers included in this review according to their publishing year.

Recent years have seen an increasing interest in using AI applications for survival prediction and risk stratification. Figure 2 shows the number of papers included in this review according to their publishing year. A variety of methods have been proposed over the past decade with progressive developments leading to current state-of-the-art methods. Older methods were based on clinical [48,49], pathological [50,51] and imaging [30,52] biomarkers, those were gradually refined with the deployment of ML and DL techniques. In this review, ML and DL algorithms are chronologically presented. Subsequently, the performance of the best performing algorithms is briefly discussed.

### 3.1. Years: 2012–2016

A series of studies focused on defining paradigms that show the potential combination of clinical and computer-aided methods. These pioneering studies laid the theoretical foundation for subsequent research based on ML and DL methods. Most of these studies focused on glioma and, in particular, GBM. However, some results can be generalised across the wide spectrum of brain tumors.

Zacharaki et al. [53] showed how predictive models, based on data mining algorithms of imaging features, provide more accurate prognostic predictions than traditional histopathological classification alone. Macyszyn et al. [54] showed how imaging patterns, analysed with with ML techniques, could provide useful information for survival prediction. Oermann et al. [55] developed an Artificial Neural Network for OS prediction that outperformed traditional statistical tools and scoring indices for individual patient prognosis prediction. Kickingereder et al. [56], in a pioneering study of radiomic profiling of GBM, identified survival-related imaging predictors that performed better than clinical and risk models. Radiomic signatures based on MRI images provided a benefit in OS prediction and risk stratification. The authors designed a Cox proportional hazard (CPH) model using radiomic features previously selected using a dimensional reduction technique. Comparing the use of radiomic features either alone or in conjunction with clinical information, they found a C-index of 0.696.

Emblem et al. [57] showed the usefulness of a SVM for SC. This model, developed particularly to perform early survival prediction in patients with glioma, showed that relative cerebral blood volume (rCBV) was the most significant imaging parameter for survival prediction. Gutman et al. [21] showed that image features, such as lesion size and enhancement after gadolinium-based contrast agent (GBCA), correlate with OS. In a pioneering work, Jain et al. [58] demonstrated a correlation between morphological features and haemodynamic parameters. This study focused on the non-enhancing tumor component and showed a significant correlation between the rCBV in the non-enhancing tumor region (NER) and the lack of epidermal growth factor receptor (EGFR) mutation.

Some studies [59,60] proposed the use of shape features extracted from the presumed-necrotic area or algorithmically assessed shape features to improve survival prediction. Liu et al. [61] proposed the use of a functional and structural brain network, by integrating information from Diffusion Tensor Imaging (DTI) and functional MRI (fMRI). Survival estimation and the subsequent subdivision into risk classes was improved by using those quantities which provided complementary information. This led to a classification accuracy of 75.0%, significantly higher than the accuracy provided by clinical information alone (accuracy = 63.2%).

These papers served as a prompt for the scientific community, and also demonstrated the potential utility of using additional information (such as radiomic, genomic, and histopathologic data [62,63]) in the predictive models rather than clinical information alone.

### 3.2. Years: 2016–2018

Research gained momentum and, while still focusing on ML and radiomics, as DL became more accessible, an increasing number of studies explored its applications in medical imaging. Several clinical, functional, radiomic and morphological biomarkers were increasingly being included in the portfolio of information used to predict survival, and found to provide added value.

Kim et al. [42] emphasized the importance of the Apparent Diffusion Coefficient (ADC) as a survival predictor biomarker, demonstrating that its significant correlation with survival. Sanghani et al. [64] showed how OS prediction is improved by using different types of radiomic features (volumetric, shape and texture) from multi-parametric MRI. They used an SVM classifier set up for stratification into 2 and 3 survival classes (short-, long-survival groups and short-, mid-, long-survival groups). The stratified 5-fold cross-validation accuracy obtained for the 2-class classifier was 97.5%, while that for the 3-class classifier was 87.1%.

Several studies highlighted that integrating multi-modal imaging and radiomic phenotyping was beneficial for OS prediction [31,56,65,66]. Bae et al. [31] trained a Random Survival Forest (RSF) model with 18 radiomic features selected by variable hunting along with clinical and genetic profiles (presence of O^6^-methylguanine-DNA-methyltransferase [MGMT] promoter methylation and isocitrate dehydrogenase 1 [IDH 1] mutation) to predict OS. The RSF model integrated with radiomic, clinical and genetic features showed the best performance (AUC = 0.74), when compared to models that only considered one type of feature. Peeken et al. [65] showed that the combination of multi-modal images was decisive for improving OS prediction; moreover, among all the considered features, those derived from MRI were the most predictive and, therefore, relevant. Particularly, for OS prediction, this model showed a C-Index of 0.61. A C-index of 0.71 was obtained by including the remaining features set and clinical information in the model. Kickingereder et al. [67] obtained a C-Index of 0.77 using a radiomic signature composed of 8 features and a Cox regression model of the least absolute shrinkage and selection operator (LASSO) penalized type. A subsequent study performed by Prasanna et al. [35] showed that the use of features from peritumoral brain parenchyma could also aid in predicting long versus short term survival. In this case, 10 features from peritumoral regions were found to be predictive, when compared with features from enhancing tumors, necrotic regions and clinical characteristics. The combination of clinical and radiomic features generated a model with C-index of 0.734, improving GBM survival prediction. Another study conducted by Kim et al. [68] showed preliminary evidence that in peritumoral NER, fractional anisotropy (FA) and normalized rCBV (nCBV) features could have improved OS prediction. OS was estimated analysing radiomic features extracted in the NER. The model, combining nCBV and FA performed better than single image/methods radiomic models and obtained a C-Index of 0.87. However, given the nature of these retrospective studies and the small sample size, the generalizability and statistical power of this data may be limited.

A few studies [36,37] explored the effect of heterogeneity on survival stratification highlighting that the distribution of heterogeneity within the tumor was a determining parameter for correct classification (the classification accuracy was in the range 78.2–80.7% [min-max]). A pioneering study [11] identified spatial image features from tumor habitats and subregions that were associated with survival time. In particular, spatial features extracted from tumor habitats were effective in predicting survival [11,69]. Two databases of GBM images were used. The model with habitat-based features for survival prediction showed promising accuracy in both databases (86.7–87.5%). Suter et al. [70] emphasise that the use of robust radiomic features could benefit the generalizability of the model, specific to OS, especially for single centre based models applied to unseen multi-centre datasets. A separate study [38] evaluated the impact of brain functional networks on OS achieving an accuracy of 86.8% using resting state fMRI (rs-fMRI) derived information. This study was based on the hypothesis that connectomics-based features could capture tumor-induced network level alterations that can influence prognosis, underlining the importance of including rs-fMRI in the pre-surgical workout of patients with glioma. Nematollahi et al. [71] showed the impact of a decision tree trained using both clinical and radiomic features. They obtained an accuracy of 90.9% for OS classification, using the C5.0 decision tree algorithm.

Preliminary results of DL applications were also beginning to emerge. Nie et al. [43] developed a DL framework for automatic extraction of features from multimodal MR images ($T_{1}$-weighted [$T_{1}$w] imaging, fMRI, DTI), combining elements of both DL (deeply learned features) and traditional ML (handcrafted features), using a tri-dimensional convolutional neural network (3D-CNN) and generating a new network architecture for using multichannel data and learning supervised features. In the long versus short time classification task (SC), i.e., dichotomous classification, the model achieved an accuracy of 89.9%. The authors particularly stressed how relevant were the features learned from DTI and fMRI.

### 3.3. Years: 2019–2020

Between 2019 and 2020, the focus shifted away from ML to pivot on DL, hybrid techniques (e.g., mixed DL + ML techniques) and CNNs, which have become one of the reference paradigms. Several studies suggested that DL-based survival prediction can outperform ML-based ones. In particular, non-linear DL methods may be useful in survival studies [72].

Way et al. [73] identified a correlation between volumetric DL features and OS. Zadeh et al. [74] developed a CNN for SC based on histopathology (DeepSurvNet) able to classify patients into 4 distinct survival classes. DeepSurvNet achieved an accuracy of 80% on blind data. Furthermore, through the analysis of mutation frequencies, DeepSurvNet was able to capture the genetic differences between the various survival classes. The use of histopathological images was therefore beneficial for SC. Nie et al. [75] used a multichannel 3D-CNN with multimodal images. Following feature extraction by DL methods, features were entered into an SVM for SC (long versus short survival). The model reported an accuracy of 90.7%.

A variety of authors have also employed hybrid techniques [44,45,76,77,78,79,80,81], often based on ML and DL. Some authors [81] have shown that by adding genomic information, the predictive accuracy would significantly increase (in this study the mean root mean square error (RMSE) was found to be reduced by 84 days compared to the use of a CNN based only on single mode MR images). Others experimented a model based on a neural network [79] to categorise survival into two classes and provide OS in days, showing inferior performance (accuracy = 0.59), therefore, indirectly justifying the use of a deep neural network, such as CNNs.

Recent studies [77] have also highlighted the usefulness of radiogenomics for OS prediction in days. A hypercolumn-based CNN was employed for segmentation, features extraction and combination of imaging-derived biomarkers with gene expression. The radiogenomic model performed best when compared to the performance of individual models with genomic and radiomic information. Of particular relevance, Zhang et al. [76] used a mixed technique to identify high-risk sub-regions within a lesion that may influence survival. In brief, K-means clustering was used for initial identification of sub-regions of interest (294 total); subsequently, a multiple-instance learning (MIL) model was used for risk stratification. The performance of high-risk regions in survival stratification showed an accuracy of 87.9%, higher than a model built using radiomic features extracted from the gross tumor region (70.19%). Different authors [44,45] focused on the impact of radiomic features on the DL model. Feng et al. [45] developed a 3D-U-Net designed to perform segmentation (since the features were designed for a segmentation task and then repurposed for a different one, accuracy in classification was not high). They used a multivariate linear regression model to minimize over fitting, although at the cost of its expressiveness. Nevertheless, the authors won the OS subtask competition at the Medical Image Computing and Computer Assisted Intervention Society Brain Tumor Segmentation (MICCAI BraTS) 2018 challenge. This paper proved the feasibility of using features not linked to a specific task. Han et al. [44], developed a mixed technique (ML + DL) able to classify patients into long- and short-term survivors with a log-rank test *P* value < 0.001.

Numerous studies [42,58,81,82] agreed on the importance of using features derived from Perfusion-Weighted Imaging (PWI) and Diffusion-Weighted Imaging (DWI). Petrova et al. [82] identified features related to ADC and rCBV parameters as possible OS predictors. In this study features were also ranked according to their importance; between ADC and rCBV features, the most important were: 95th percentile values for ADC (ADC_95), standard deviation of rCBV (rCBV_std), standard deviation of ADC (ADC_std), and median of rCBV (rCBV_median). Sun et al. [83] presented a DL-based framework for brain tumor segmentation and survival prediction in glioma, using multimodal MRI scans. Ensembles of three different 3D CNN architectures for robust performance through a majority rule were used for tumor segmentation. For survival prediction, 4524 radiomic features were extracted from segmented tumor regions, then, a decision tree and cross validation were used to select relevant features. Finally, a random forest model was trained to predict OS. This method ranked 5th at the MICCAI BraTS 2018, with 61.0% accuracy for classification in short-, mid- or long-survivors.

Several studies [59,73,78,84,85,86,87,88] have shown a potential correlation of radiomic features extracted from MRI with OS, which is emerging to be helpful in predicting GBM OS. Lu et al. [87] developed a ML model for predicting OS in GBM, based on the use of radiomic, clinical and semantic features, the latter based on the Visually AccesSAble Rembrandt Images (VASARI) feature scoring system. This study, based on contrast-enhanced $T_{1}$-weighted (CE-$T_{1}$w) imaging, showed excellent performance for OS prediction. 333 radiomic features and 16 semantic features (VASARI) were extracted; following the selection and ranking of radiomic features, together with semantic and clinical features, the authors built a ML model aimed primarily at predicting MGMT promoter methylation status. MGMT methylation was used with the previously determined set of features (radiomic, clinical and semantic) to build a second model to predict OS. Both a CPH regression model and a RSF model were tested. The RSF model had the best performance, with C-Index of 0.91.

### 3.4. Years: 2021–2022

More recently, we observed a consolidation in DL applications such as CNN and, often, hybrid methods consisting of ML and radiomics.

Chen et al. [89] hypothesised that combining dose-volume histogram (DVH) and clinical features into a single model could lead to better performance than using clinical features alone. Thus, they developed an ML-based model integrating clinical and dose volume histograms parameters demonstrating that this integration can improve risk modelling. They also compared the performance of RSF and CPH to individuate the best classifier. RSF performed better on the testing set, with a C-Index of 0.85. The RSF-based model obtained AUC values of 0.91, 0.88 and 0.84 in predicting survival at 1, 2, and 3 years respectively, therefore showing good predictive accuracy. Gross tumor volume (GTV) and D99 (also called the near- minimum absorbed dose, represents the dose that covers 99% of the target volume) features were also identified as potential new diagnostic biomarkers, in addition to presence of IDH 1 mutation, Karnofsky performance status (KPS) and smoking status. Rathore et al. [90] showed that combining in a classifier both MRI, radiomic and histopathological imaging features can be beneficial for OS prediction, compared to the performance of classifiers based on a single feature type (MRI, radiomic or histopathological) only. The accuracy in predicting survival in groups was 0.86, while the C-Index was 0.79.

Huang et al. [40] developed a method that allows prediction of survival with random forest regressors. A V-Net was used for feature extraction, mainly focused on segmentation tasks. This project presented an integrated framework between segmentation and survival prediction. It also achieved an average RMSE of 311.5 for survival prediction, outperforming other methods proposed by other participants during the BraTS competition. Wang et al. [91] developed a radiomic signature as pre-treatment predictor of OS. A radiomic signature derived from CE-$T_{1}$w and FLAIR sequences, showed better prognostic performance than signature derived from either individual imaging techniques, and obtained a C-Index of 0.798, out-performing the use of clinical and pathological information only, which obtained a C-Index of 0.675. According to those results, the radiomic signature may help to identify patients who would benefit from chemotherapy. The study identified patients with low grade glioma (LGG) who may have worse survival, and, thanks to the radiomic signature, they selected patients who may benefit the most from temozolomide (TMZ).

Although slightly inferior in performance, the approach published by Preetha et al. [39], may have a potentially significant clinical impact by reducing the necessity of contrast administration for serial scans. The authors generated post-contrast $T_{1}$w synthetic MR images from pre-contrast $T_{1}$w MR images using a deep CNN (dCNN). The quantification of the contrast-enhancing area from post-contrast synthetic $T_{1}$w MRI allowed assessment of the patient’s response to treatment without any significant difference from the true post-contrast $T_{1}$w sequences obtained after GBCA administration. These promising results could promote the application of dCNN in radiology to potentially reduce the need for GBCA administration. The authors did not observe any significant difference in OS estimated using the original or the synthetically obtained images. The synthetic images showed a C-Index of 0.667, while the original images show a C-Index of 0.673.

Various authors focused on the combined use of ML and radiomic techniques for OS prediction in brain tumors [92,93]. Chato et al. [92] focused on GBM, while Grist et al. [93] focused on paediatric brain tumors. The latter combined multi-site MRI with ML methods to predict survival in paediatric brain tumors, with the aim of stratifying patients to low and high risk cohorts. In Grist et al. [93], patients underwent PWI and DWI at the time of diagnosis. After conventional post-processing, a semi-supervised Bayesian survival analysis was performed. Unsupervised and supervised ML were then performed to determine sub-groups with different survival, and assess subsequent classification accuracy. A combination of DWI and PWI was able to determine two sub-groups of brain tumors with different survival characteristics. Kaplan–Meier analysis of high-grade tumors in the high and low risk clusters revealed a significant difference in survival characteristics (p<0.05), which were subsequently classified with high accuracy (98%) by a single layer Neural Network, after stratified ten-fold cross validation. The same task with a logistic regressor generated an accuracy of 90%, indicating that the neural network was more suitable for this type of task. The model-relevant features, were: uncorrected Cerebral Blood Volume (uCBV), Region of Interest (ROI) mean, a vascular leakage parameter (K2) ROI mean, uCBV whole brain mean, tumor volume, and ADC ROI kurtosis. Tumor perfusion measures were found to be of high importance in determining survival. The authors also state that perfusion was so relevant for classification purposes that it should be included in clinical imaging protocols. A particular strength of this work was that it was performed on multi-site, multi-scanner data.

### 3.5. Overall Considerations

The main limitations we identified in the reviewed papers are related to the improper use of techniques and algorithms (often not state-of-the-art), the use of limited or sub-optimal datasets (e.g., different image acquisition parameters, lack of standardization protocols or incomplete information) and the use of retrospective cohorts. The combination of these factors had a major impact on the performance of the different methods.

### 3.6. Performance

**Figure 3:**
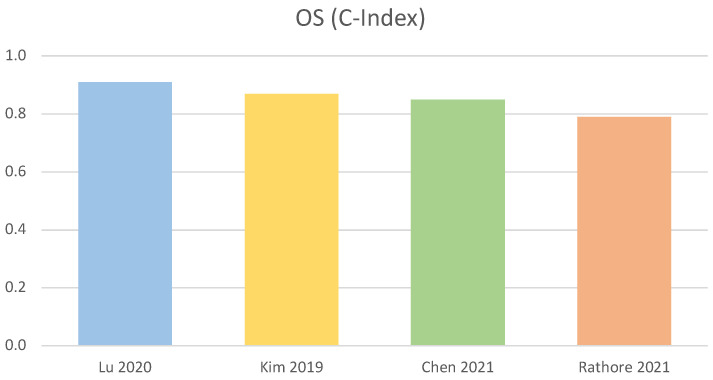
Performance of OS task: Graphical representation of the performance of the four best methods for the Overall Survival (OS) task, as evaluated by C-Index [68,87,89,90].

Table 1 summarises characteristics and performance of the best four methods for OS prediction. Among those, the C-Index fluctuates from 0.79 [90] to 0.91 [87], with the best performance obtained by an RSF. A graphical representation of the C-Index of the best four methods is displayed in Figure 3.

**Figure 4:**
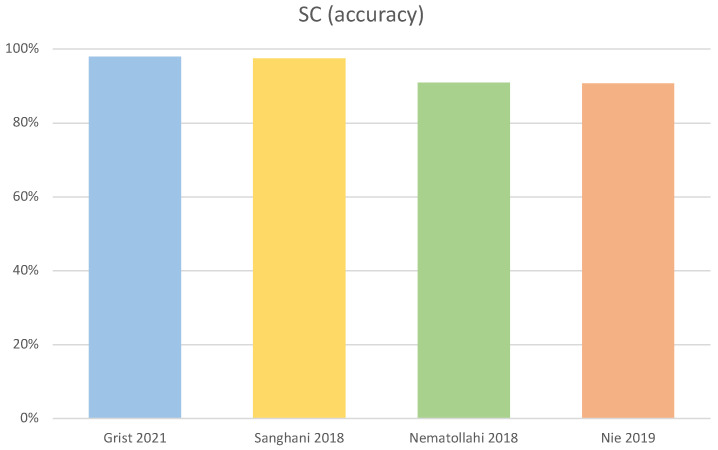
Performance of SC task: Graphical representation of the performance of the four best methods for the Survival Classification (SC) task, as evaluated by accuracy [64,71,75,93].

For the SC task, Single Layer Neural Network [93], SVM [64], Decision Trees [71], and 3D-CNN plus a SVM [75] achieved the highest accuracy (>90.7%). Characteristics and performance of those four methods are summarised in Table 2, a graphical representation of the accuracy of the best four methods is displayed in Figure 4.

## 4. Discussion

We presented a general overview of the current literature on AI applications in predicting survival in patients with brain tumors, based on MRI. The use of AI-based techniques, such as ML and DL, appears beneficial to predict survival. After evaluating several applications, we ranked the best applications based on the performance of the different algorithms for the two tasks of interest (OS and SC). Different approaches showed high performance, and the choice of the best one to use is non-univocal and subject to different variables. Unequivocally, the use of features derived from PWI and DWI/DTI were of significant relevance for both tasks. Indeed, the use of quantitative imaging is undoubtedly advantageous for AI applications. This is of particular relevance in an era when fully-quantitative MR imaging methods are becoming increasingly available and proven to be reproducible across different vendors [94].

The use of semantic features in addition to clinical and radiomic features proved of significant relevance for the OS task, similarly to the use of features from clinicopathological information. The use of multiparametric MR images, compared to unimodal ones, also leads to significant improvements. ML methods appeared to perform better for this task. The best four algorithms have C-Index values in the range 0.79–0.91. The best algorithms were ML methods employing: radiomic, clinical and semantic features [87], PWI and DTI features [68], clinical features and DVH features [89], multimodal imaging and histopathological information [90]. For SC, the best performance was shown by: a Single Layer Neural network used in conjunction with PWI and DWI features, and a SVM classifier trained with volumetric, texture and shape features extracted from multimodal MR images. Both achieved accuracy in the order of 98%. The methods using CNN and decision trees performed slightly worse. Overall, the best four algorithms have accuracy values in the range 90.7–98.0%. More in details, the best performance was shown by a Single Layer Neural Network using PWI and DWI features [93], followed by SVM with volumetric, texture, shape features and multimodal imaging [64], a decision tree-based method [71], and a DL method, based on a 3D-CNN [75].

It is also worth noting that state-of-the-art methods and algorithms may not be those used in international competitions (e.g., MICCAI BraTS) which may have intrinsic limitations. For instance, BraTS is an international challenge focused on tumor segmentation and not OS classification (which was only a subchallenge); therefore the selected features were not necessarily optimised for OS. Most of the methods presented in this context, either based on a typical ML or DL architecture, extract and use significant features to achieve the best possible segmentation (the primary task) and often employ these features also for the secondary task (OS prediction). Hence, OS prediction is performed on the features that were chosen to obtain the best possible segmentation, without building a model focused on OS prediction itself. Therefore, those non-optimised models may obtain worse performance than state-of-the-art methods exclusively focused on survival prediction.

## 5. Conclusions

In conclusion, depending on the specific task, different algorithms perform differently. In particular, ML methods, integrated with additional information, including clinical, radiomic, semantic and DWI/PWI information showed the best performances for OS prediction. Without the use of this additional information, DL methods would have performed better.

Future studies should focus on developing ML/DL models by combining different data sources (i.e., clinical, radiomic, semantic and PWI/DWI), which are correlated and may provide complementary information [95] for improving the clinical decision-making tasks [96]. Moreover, given the proven importance of quantitative techniques such as PWI and DWI, future ML/DL models should leverage the wealth of data provided by novel and more refined diffusion and perfusion techniques [97,98,99,100,101], potentially also including information upon cerebral metabolism [99,102]. By integrating all these data into a single multimodal model, further improvements in performance could be achieved. Lastly, the research community should also plan to evaluate the impact of these integrative DL-based models, and compare their performance against analogous ML-based models.

## Figures and Tables

**Table 1:** Characteristics and performance (C-Index) of the four methods with the highest C-Index for Overall Survival (OS).

Overall Survival (OS)					
**Reference**	**AI Method**	**Evaluation Procedure**	**Number of Cases**	**Analysed Features**	**Performance on Test Set (C-Index)**
Lu et al. [87] (2020)	Random Survival Forest (RSF)	70–30% + 10-fold CV	181	Radiomic, Clinical, Semantic (VASARI) features	0.91
Kim et al. [68] (2019)	Generalised Linear Model	70–30% + 10-fold CV + LASSO	83	Radiomic, Clinical, PWI, DTI features	0.87
Chen et al. [89] (2021)	Random Survival Forest (RSF)	60–40%	95	Clinical, DVH features	0.85
Rathore et al. [90] (2021)	Cox Proportional Hazard Regression (CPH)	60–40%	171	Radiomic, histopathological features + Multimodal imaging	0.79

**Table 2:** Characteristics and performance (accuracy) of the four methods with the highest accuracy for Survival Classification (SC).

Survival Classification (SC)					
**Reference**	**AI Method**	**Evaluation Procedure**	**Number of Cases**	**Analysed Features**	**Performance on Test Set (Accuracy)**
Grist et al. [93] (2021)	Single Layer Neural Network	Stratified 10-fold CV	69	Clinical, Bayesian, PWI, DWI features	98.0%
Sanghani et al. [64] (2018)	Support Vector Machine (SVM)	Stratified 5-fold CV	163	Radiomic, Volumetric features + Multimodal imaging	97.5%
Nematollahi et al. [71] (2018)	Decision Trees	10-fold CV	55	Clinical, Imaging (MRI) features + Multimodal imaging	90.9%
Nie et al. [75] (2019)	3D − Convolutional Neural Network (CNN) + SVM	75–25% + 3-fold CV	68	Deeply learned (DTI, fMRI) features + Multimodal imaging	90.7%

## Data Availability

Not applicable.

## References

[1] Castiglioni I., Rundo L., Codari M., Di Leo G., Salvatore C., Interlenghi M., Gallivanone F., Cozzi A., D’Amico N.C., Sardanelli F. (2021). AI applications to medical images: From machine learning to deep learning. Phys. Med..

[2] Segato A., Marzullo A., Calimeri F., De Momi E. (2020). Artificial intelligence for brain diseases: A systematic review. APL Bioeng..

[3] Senders J.T., Arnaout O., Karhade A.V., Dasenbrock H.H., Gormley W.B., Broekman M.L., Smith T.R. (2018). Natural and artificial intelligence in neurosurgery: A systematic review. Neurosurgery.

[4] Senders J.T., Staples P.C., Karhade A.V., Zaki M.M., Gormley W.B., Broekman M.L., Smith T.R., Arnaout O. (2018). Machine learning and neurosurgical outcome prediction: A systematic review. World Neurosurg..

[5] Alhasan A.S. (2021). Clinical Applications of Artificial Intelligence, Machine Learning, and Deep Learning in the Imaging of Gliomas: A Systematic Review. Cureus.

[6] Siegel R.L., Miller K.D., Fuchs H.E., Jemal A. (2021). Cancer Statistics, 2021. CA Cancer J. Clin..

[7] Yi Z., Long L., Zeng Y., Liu Z. (2021). Current Advances and Challenges in Radiomics of Brain Tumors. Front. Oncol..

[8] Ostrom Q.T., Gittleman H., Fulop J., Liu M., Blanda R., Kromer C., Wolinsky Y., Kruchko C., Barnholtz-Sloan J.S. (2015). CBTRUS statistical report: Primary brain and central nervous system tumors diagnosed in the United States in 2008–2012. Neuro-Oncology.

[9] Louis D.N., Perry A., Wesseling P., Brat D.J., Cree I.A., Figarella-Branger D., Hawkins C., Ng H., Pfister S.M., Reifenberger G. (2021). The 2021 WHO classification of tumors of the central nervous system: A summary. Neuro-Oncology.

[10] Belden C.J., Valdes P.A., Ran C., Pastel D.A., Harris B.T., Fadul C.E., Israel M.A., Paulsen K., Roberts D.W. (2011). Genetics of glioblastoma: A window into its imaging and histopathologic variability. Radiographics.

[11] Zhou M., Chaudhury B., Hall L.O., Goldgof D.B., Gillies R.J., Gatenby R.A. (2017). Identifying spatial imaging biomarkers of glioblastoma multiforme for survival group prediction. J. Magn. Reson. Imaging.

[12] Ostrom Q.T. (2015). Epidemiology of Gliomas. Current Understanding and Treatment of Gliomas.

[13] Miller K.D., Ostrom Q.T., Kruchko C., Patil N., Tihan T., Cioffi G., Fuchs H.E., Waite K.A., Jemal A., Siegel R.L. (2021). Brain and other central nervous system tumor statistics, 2021. CA Cancer J. Clin..

[14] Tykocki T., Eltayeb M. (2018). Ten-year survival in glioblastoma. A systematic review. J. Clin. Neurosci..

[15] Delgado-López P., Corrales-García E. (2016). Survival in glioblastoma: A review on the impact of treatment modalities. Clin. Transl. Oncol..

[16] Ostrom Q.T., Gittleman H., Truitt G., Boscia A., Kruchko C., Barnholtz-Sloan J.S. (2018). CBTRUS statistical report: Primary brain and other central nervous system tumors diagnosed in the United States in 2011–2015. Neuro-Oncology.

[17] Tan A.C., Ashley D.M., López G.Y., Malinzak M., Friedman H.S., Khasraw M. (2020). Management of glioblastoma: State of the art and future directions. CA Cancer J. Clin..

[18] Omuro A., DeAngelis L.M. (2013). Glioblastoma and other malignant gliomas: A clinical review. JAMA.

[19] Krex D., Klink B., Hartmann C., Von Deimling A., Pietsch T., Simon M., Sabel M., Steinbach J.P., Heese O., Reifenberger G. (2007). Long-term survival with glioblastoma multiforme. Brain.

[20] Zaccagna F., Grist J.T., Quartuccio N., Riemer F., Fraioli F., Caracò C., Halsey R., Aldalilah Y., Cunningham C.H., Massoud T.F. (2021). Imaging and treatment of brain tumors through molecular targeting: Recent clinical advances. Eur. J. Radiol..

[21] Gutman D.A., Cooper L.A., Hwang S.N., Holder C.A., Gao J., Aurora T.D., Dunn Jr W.D., Scarpace L., Mikkelsen T., Jain R. (2013). MR imaging predictors of molecular profile and survival: Multi-institutional study of the TCGA glioblastoma data set. Radiology.

[22] Villanueva-Meyer J.E., Mabray M.C., Cha S. (2017). Current clinical brain tumor imaging. Neurosurgery.

[23] Langen K.J., Galldiks N., Hattingen E., Shah N.J. (2017). Advances in neuro-oncology imaging. Nat. Rev. Neurol..

[24] Kim M.M., Parolia A., Dunphy M.P., Venneti S. (2016). Non-invasive metabolic imaging of brain tumours in the era of precision medicine. Nat. Rev. Clin. Oncol..

[25] Zaccagna F., Grist J.T., Deen S.S., Woitek R., Lechermann L.M., McLean M.A., Basu B., Gallagher F.A. (2018). Hyperpolarized carbon-13 magnetic resonance spectroscopic imaging: A clinical tool for studying tumour metabolism. Br. J. Radiol..

[26] Aerts H.J., Velazquez E.R., Leijenaar R.T., Parmar C., Grossmann P., Carvalho S., Bussink J., Monshouwer R., Haibe-Kains B., Rietveld D. (2014). Decoding tumour phenotype by noninvasive imaging using a quantitative radiomics approach. Nat. Commun..

[27] Yip S.S., Aerts H.J. (2016). Applications and limitations of radiomics. Phys. Med. Biol..

[28] Gillies R.J., Kinahan P.E., Hricak H. (2016). Radiomics: Images are more than pictures, they are data. Radiology.

[29] Theek B., Opacic T., Magnuska Z., Lammers T., Kiessling F. (2018). Radiomic analysis of contrast-enhanced ultrasound data. Sci. Rep..

[30] Zaccagna F., Ganeshan B., Arca M., Rengo M., Napoli A., Rundo L., Groves A.M., Laghi A., Carbone I., Menezes L.J. (2021). CT texture-based radiomics analysis of carotid arteries identifies vulnerable patients: A preliminary outcome study. Neuroradiology.

[31] Bae S., Choi Y.S., Ahn S.S., Chang J.H., Kang S.G., Kim E.H., Kim S.H., Lee S.K. (2018). Radiomic MRI phenotyping of glioblastoma: Improving survival prediction. Radiology.

[32] Hassani C., Saremi F., Varghese B.A., Duddalwar V. (2020). Myocardial radiomics in cardiac MRI. Am. J. Roentgenol..

[33] Jang J., El-Rewaidy H., Ngo L.H., Mancio J., Csecs I., Rodriguez J., Pierce P., Goddu B., Neisius U., Manning W. (2021). Sensitivity of myocardial radiomic features to imaging parameters in cardiac MR imaging. J. Magn. Reson. Imaging.

[34] Wang J., Bravo L., Wan K., Sun J., Zhu Y., Han Y., Gkoutos G.V., Chen Y. (2021). Radiomics analysis derived from LGE-MRI predict sudden cardiac death in participants with hypertrophic cardiomyopathy. Front. Cardiovasc. Med..

[35] Prasanna P., Patel J., Partovi S., Madabhushi A., Tiwari P. (2017). Radiomic features from the peritumoral brain parenchyma on treatment-naive multi-parametric MR imaging predict long versus short-term survival in glioblastoma multiforme: Preliminary findings. Eur. Radiol..

[36] Liu Y., Xu X., Yin L., Zhang X., Li L., Lu H. (2017). Relationship between glioblastoma heterogeneity and survival time: An MR imaging texture analysis. Am. J. Neuroradiol..

[37] Liu Y., Zhang X., Feng N., Yin L., He Y., Xu X., Lu H. (2018). The effect of glioblastoma heterogeneity on survival stratification: A multimodal MR imaging texture analysis. Acta Radiol..

[38] Liu L., Zhang H., Wu J., Yu Z., Chen X., Rekik I., Wang Q., Lu J., Shen D. (2019). Overall survival time prediction for high-grade glioma patients based on large-scale brain functional networks. Brain Imaging Behav..

[39] Preetha C.J., Meredig H., Brugnara G., Mahmutoglu M.A., Foltyn M., Isensee F., Kessler T., Pflüger I., Schell M., Neuberger U. (2021). Deep-learning-based synthesis of post-contrast T1-weighted MRI for tumour response assessment in neuro-oncology: A multicentre, retrospective cohort study. Lancet Digit. Health.

[40] Huang H., Zhang W., Fang Y., Hong J., Su S., Lai X. (2021). Overall Survival Prediction for Gliomas Using a Novel Compound Approach. Front. Oncol..

[41] Bakas S., Shukla G., Akbari H., Erus G., Sotiras A., Rathore S., Sako C., Ha S.M., Rozycki M., Shinohara R.T. (2020). Overall survival prediction in glioblastoma patients using structural magnetic resonance imaging (MRI): Advanced radiomic features may compensate for lack of advanced MRI modalities. J. Med. Imaging.

[42] Kim B.S., Kim S.T., Kim J.H., Seol H.J., Nam D.H., Shin H.J., Lee J.I., Kong D.S. (2019). Apparent diffusion coefficient as a predictive biomarker for survival in patients with treatment-naive glioblastoma using quantitative multiparametric magnetic resonance profiling. World Neurosurg..

[43] Nie D., Zhang H., Adeli E., Liu L., Shen D. 3D deep learning for multi-modal imaging-guided survival time prediction of brain tumor patients. Proceedings of the International Conference on Medical Image Computing and Computer-Assisted Intervention.

[44] Han W., Qin L., Bay C., Chen X., Yu K.H., Miskin N., Li A., Xu X., Young G. (2020). Deep transfer learning and radiomics feature prediction of survival of patients with high-grade gliomas. Am. J. Neuroradiol..

[45] Feng X., Tustison N.J., Patel S.H., Meyer C.H. (2020). Brain tumor segmentation using an ensemble of 3d u-nets and overall survival prediction using radiomic features. Front. Comput. Neurosci..

[46] Kamnitsas K., Ferrante E., Parisot S., Ledig C., Nori A.V., Criminisi A., Rueckert D., Glocker B. DeepMedic for brain tumor segmentation. Proceedings of the International Workshop on Brainlesion: Glioma, Multiple Sclerosis, Stroke and Traumatic Brain Injuries.

[47] Chato L., Latifi S. Machine learning and deep learning techniques to predict overall survival of brain tumor patients using MRI images. Proceedings of the 2017 IEEE 17th International Conference on Bioinformatics and Bioengineering (BIBE).

[48] Hamilton W., Kernick D. (2007). Clinical features of primary brain tumours: A case–control study using electronic primary care records. Br. J. Gen. Pract..

[49] Kane A.J., Sughrue M.E., Rutkowski M.J., Shangari G., Fang S., McDermott M.W., Berger M.S., Parsa A.T. (2011). Anatomic location is a risk factor for atypical and malignant meningiomas. Cancer.

[50] Ideguchi M., Kajiwara K., Goto H., Sugimoto K., Nomura S., Ikeda E., Suzuki M. (2015). MRI findings and pathological features in early-stage glioblastoma. J. Neuro-Oncol..

[51] Heynold E., Zimmermann M., Hore N., Buchfelder M., Doerfler A., Stadlbauer A., Kremenevski N. (2021). Physiological MRI Biomarkers in the Differentiation Between Glioblastomas and Solitary Brain Metastases. Mol. Imaging Biol..

[52] Galanaud D., Nicoli F., Chinot O., Confort-Gouny S., Figarella-Branger D., Roche P., Fuentès S., Le Fur Y., Ranjeva J.P., Cozzone P.J. (2006). Noninvasive diagnostic assessment of brain tumors using combined in vivo MR imaging and spectroscopy. Magn. Reson. Med. Off. J. Int. Soc. Magn. Reson. Med..

[53] Zacharaki E.I., Morita N., Bhatt P., O’rourke D., Melhem E., Davatzikos C. (2012). Survival analysis of patients with high-grade gliomas based on data mining of imaging variables. Am. J. Neuroradiol..

[54] Macyszyn L., Akbari H., Pisapia J.M., Da X., Attiah M., Pigrish V., Bi Y., Pal S., Davuluri R.V., Roccograndi L. (2015). Imaging patterns predict patient survival and molecular subtype in glioblastoma via machine learning techniques. Neuro-Oncology.

[55] Oermann E.K., Kress M.A.S., Collins B.T., Collins S.P., Morris D., Ahalt S.C., Ewend M.G. (2013). Predicting survival in patients with brain metastases treated with radiosurgery using artificial neural networks. Neurosurgery.

[56] Kickingereder P., Burth S., Wick A., Götz M., Eidel O., Schlemmer H.P., Maier-Hein K.H., Wick W., Bendszus M., Radbruch A. (2016). Radiomic profiling of glioblastoma: Identifying an imaging predictor of patient survival with improved performance over established clinical and radiologic risk models. Radiology.

[57] Emblem K.E., Pinho M.C., Zöllner F.G., Due-Tonnessen P., Hald J.K., Schad L.R., Meling T.R., Rapalino O., Bjornerud A. (2015). A generic support vector machine model for preoperative glioma survival associations. Radiology.

[58] Jain R., Poisson L.M., Gutman D., Scarpace L., Hwang S.N., Holder C.A., Wintermark M., Rao A., Colen R.R., Kirby J. (2014). Outcome prediction in patients with glioblastoma by using imaging, clinical, and genomic biomarkers: Focus on the nonenhancing component of the tumor. Radiology.

[59] Chaddad A., Desrosiers C., Hassan L., Tanougast C. (2016). A quantitative study of shape descriptors from glioblastoma multiforme phenotypes for predicting survival outcome. Br. J. Radiol..

[60] Czarnek N., Clark K., Peters K.B., Mazurowski M.A. (2017). Algorithmic three-dimensional analysis of tumor shape in MRI improves prognosis of survival in glioblastoma: A multi-institutional study. J. Neuro-Oncol..

[61] Liu L., Zhang H., Rekik I., Chen X., Wang Q., Shen D. Outcome prediction for patient with high-grade gliomas from brain functional and structural networks. Proceedings of the International Conference on Medical Image Computing and Computer-Assisted Intervention.

[62] Tan Y., Mu W., Wang X.C., Yang G.Q., Gillies R.J., Zhang H. (2019). Improving survival prediction of high-grade glioma via machine learning techniques based on MRI radiomic, genetic and clinical risk factors. Eur. J. Radiol..

[63] Ammari S., Sallé de Chou R., Balleyguier C., Chouzenoux E., Touat M., Quillent A., Dumont S., Bockel S., Garcia G.C., Elhaik M. (2021). A Predictive Clinical-Radiomics Nomogram for Survival Prediction of Glioblastoma Using MRI. Diagnostics.

[64] Sanghani P., Ang B.T., King N.K.K., Ren H. (2018). Overall survival prediction in glioblastoma multiforme patients from volumetric, shape and texture features using machine learning. Surg. Oncol..

[65] Peeken J.C., Goldberg T., Pyka T., Bernhofer M., Wiestler B., Kessel K.A., Tafti P.D., Nüsslin F., Braun A.E., Zimmer C. (2019). Combining multimodal imaging and treatment features improves machine learning-based prognostic assessment in patients with glioblastoma multiforme. Cancer Med..

[66] Choi Y.S., Ahn S.S., Chang J.H., Kang S.G., Kim E.H., Kim S.H., Jain R., Lee S.K. (2020). Machine learning and radiomic phenotyping of lower grade gliomas: Improving survival prediction. Eur. Radiol..

[67] Kickingereder P., Neuberger U., Bonekamp D., Piechotta P.L., Götz M., Wick A., Sill M., Kratz A., Shinohara R.T., Jones D.T. (2018). Radiomic subtyping improves disease stratification beyond key molecular, clinical, and standard imaging characteristics in patients with glioblastoma. Neuro-Oncology.

[68] Kim J.Y., Yoon M.J., Park J.E., Choi E.J., Lee J., Kim H.S. (2019). Radiomics in peritumoral non-enhancing regions: Fractional anisotropy and cerebral blood volume improve prediction of local progression and overall survival in patients with glioblastoma. Neuroradiology.

[69] Verma R., Correa R., Hill V.B., Statsevych V., Bera K., Beig N., Mahammedi A., Madabhushi A., Ahluwalia M., Tiwari P. (2020). Tumor habitat–derived radiomic features at pretreatment MRI that are prognostic for progression-free survival in glioblastoma are associated with key morphologic attributes at histopathologic examination: A feasibility study. Radiol. Artif. Intell..

[70] Suter Y., Knecht U., Alão M., Valenzuela W., Hewer E., Schucht P., Wiest R., Reyes M. (2020). Radiomics for glioblastoma survival analysis in pre-operative MRI: Exploring feature robustness, class boundaries, and machine learning techniques. Cancer Imaging.

[71] Nematollahi M., Jajroudi M., Arbabi F., Azarhomayoun A., Azimifar Z. (2018). The benefits of decision tree to predict survival in patients with glioblastoma multiforme with the use of clinical and imaging features. Asian J. Neurosurg..

[72] Bice N., Kirby N., Bahr T., Rasmussen K., Saenz D., Wagner T., Papanikolaou N., Fakhreddine M. (2020). Deep learning-based survival analysis for brain metastasis patients with the national cancer database. J. Appl. Clin. Med. Phys..

[73] Wan Y., Rahmat R., Price S.J. (2020). Deep learning for glioblastoma segmentation using preoperative magnetic resonance imaging identifies volumetric features associated with survival. Acta Neurochir..

[74] Zadeh Shirazi A., Fornaciari E., Bagherian N.S., Ebert L.M., Koszyca B., Gomez G.A. (2020). DeepSurvNet: Deep survival convolutional network for brain cancer survival rate classification based on histopathological images. Med. Biol. Eng. Comput..

[75] Nie D., Lu J., Zhang H., Adeli E., Wang J., Yu Z., Liu L., Wang Q., Wu J., Shen D. (2019). Multi-channel 3D deep feature learning for survival time prediction of brain tumor patients using multi-modal neuroimages. Sci. Rep..

[76] Zhang X., Lu D., Gao P., Tian Q., Lu H., Xu X., He X., Liu Y. (2020). Survival-relevant high-risk subregion identification for glioblastoma patients: The MRI-based multiple instance learning approach. Eur. Radiol..

[77] Wijethilake N., Islam M., Ren H. (2020). Radiogenomics model for overall survival prediction of glioblastoma. Med Biol. Eng. Comput..

[78] Luo H., Zhuang Q., Wang Y., Abudumijiti A., Shi K., Rominger A., Chen H., Yang Z., Tran V., Wu G. (2021). A novel image signature-based radiomics method to achieve precise diagnosis and prognostic stratification of gliomas. Lab. Investig..

[79] Baid U., Rane S.U., Talbar S., Gupta S., Thakur M.H., Moiyadi A., Mahajan A. (2020). Overall survival prediction in glioblastoma with radiomic features using machine learning. Front. Comput. Neurosci..

[80] Pei L., Vidyaratne L., Rahman M.M., Iftekharuddin K.M. (2020). Context aware deep learning for brain tumor segmentation, subtype classification, and survival prediction using radiology images. Sci. Rep..

[81] Tang Z., Xu Y., Jin L., Aibaidula A., Lu J., Jiao Z., Wu J., Zhang H., Shen D. (2020). Deep learning of imaging phenotype and genotype for predicting overall survival time of glioblastoma patients. IEEE Trans. Med Imaging.

[82] Petrova L., Korfiatis P., Petr O., LaChance D.H., Parney I., Buckner J.C., Erickson B.J. (2019). Cerebral blood volume and apparent diffusion coefficient–Valuable predictors of non-response to bevacizumab treatment in patients with recurrent glioblastoma. J. Neurol. Sci..

[83] Sun L., Zhang S., Chen H., Luo L. (2019). Brain tumor segmentation and survival prediction using multimodal MRI scans with deep learning. Front. Neurosci..

[84] Xi Y.B., Guo F., Xu Z.L., Li C., Wei W., Tian P., Liu T.T., Liu L., Chen G., Ye J. (2018). Radiomics signature: A potential biomarker for the prediction of MGMT promoter methylation in glioblastoma. J. Magn. Reson. Imaging.

[85] Korfiatis P., Kline T.L., Coufalova L., Lachance D.H., Parney I.F., Carter R.E., Buckner J.C., Erickson B.J. (2016). MRI texture features as biomarkers to predict MGMT methylation status in glioblastomas. Med. Phys..

[86] Zhou J., Reddy M., Wilson B., Blair D., Taha A., Frampton C., Eiholzer R., Gan P., Ziad F., Thotathil Z. (2018). MR imaging characteristics associate with tumor-associated macrophages in glioblastoma and provide an improved signature for survival prognostication. Am. J. Neuroradiol..

[87] Lu Y., Patel M., Natarajan K., Ughratdar I., Sanghera P., Jena R., Watts C., Sawlani V. (2020). Machine learning-based radiomic, clinical and semantic feature analysis for predicting overall survival and MGMT promoter methylation status in patients with glioblastoma. Magn. Reson. Imaging.

[88] Wu G., Shi Z., Chen Y., Wang Y., Yu J., Lv X., Chen L., Ju X., Chen Z. (2019). A sparse representation-based radiomics for outcome prediction of higher grade gliomas. Med. Phys..

[89] Chen H., Li C., Zheng L., Lu W., Li Y., Wei Q. (2021). A machine learning-based survival prediction model of high grade glioma by integration of clinical and dose-volume histogram parameters. Cancer Med..

[90] Rathore S., Chaddad A., Iftikhar M.A., Bilello M., Abdulkadir A. (2021). Combining MRI and Histologic Imaging Features for Predicting Overall Survival in Patients with Glioma. Radiol. Imaging Cancer.

[91] Wang J., Zheng X., Zhang J., Xue H., Wang L., Jing R., Chen S., Che F., Heng X., Li G. (2021). An MRI-based radiomics signature as a pretreatment noninvasive predictor of overall survival and chemotherapeutic benefits in lower-grade gliomas. Eur. Radiol..

[92] Chato L., Latifi S. (2021). Machine Learning and Radiomic Features to Predict Overall Survival Time for Glioblastoma Patients. J. Pers. Med..

[93] Grist J.T., Withey S., Bennett C., Rose H.E., MacPherson L., Oates A., Powell S., Novak J., Abernethy L., Pizer B. (2021). Combining multi-site magnetic resonance imaging with machine learning predicts survival in pediatric brain tumors. Sci. Rep..

[94] Buonincontri G., Biagi L., Retico A., Cecchi P., Cosottini M., Gallagher F.A., Gómez P.A., Graves M.J., McLean M.A., Riemer F. (2019). Multi-site repeatability and reproducibility of MR fingerprinting of the healthy brain at 1.5 and 3.0 T. NeuroImage.

[95] Rundo L., Militello C., Vitabile S., Russo G., Sala E., Gilardi M.C. (2020). A survey on nature-inspired medical image analysis: A step further in biomedical data integration. Fundam. Inform..

[96] Rundo L., Pirrone R., Vitabile S., Sala E., Gambino O. (2020). Recent advances of HCI in decision-making tasks for optimized clinical workflows and precision medicine. J. Biomed. Inform..

[97] Starck L., Zaccagna F., Pasternak O., Gallagher F.A., Grüner R., Riemer F. (2021). Effects of Multi-Shell Free Water Correction on Glioma Characterization. Diagnostics.

[98] Zaccagna F., Riemer F., Priest A.N., McLean M.A., Allinson K., Grist J.T., Dragos C., Matys T., Gillard J.H., Watts C. (2019). Non-invasive assessment of glioma microstructure using VERDICT MRI: Correlation with histology. Eur. Radiol..

[99] Grist J.T., Miller J.J., Zaccagna F., McLean M.A., Riemer F., Matys T., Tyler D.J., Laustsen C., Coles A.J., Gallagher F.A. (2020). Hyperpolarized 13C MRI: A novel approach for probing cerebral metabolism in health and neurological disease. J. Cereb. Blood Flow Metab..

[100] Flies C.M., Snijders T.J., Van Seeters T., Smits M., De Vos F.Y., Hendrikse J., Dankbaar J.W. (2021). Perfusion imaging with arterial spin labeling (ASL)–MRI predicts malignant progression in low-grade (WHO grade II) gliomas. Neuroradiology.

[101] Testud B., Brun G., Varoquaux A., Hak J., Appay R., Le Troter A., Girard N., Stellmann J. (2021). Perfusion-weighted techniques in MRI grading of pediatric cerebral tumors: Efficiency of dynamic susceptibility contrast and arterial spin labeling. Neuroradiology.

[102] Zaccagna F., McLean M., Grist J., Kaggie J., Mair R., Riemer F., Woitek R., Gill A., Deen S., Daniels C. (2022). Imaging Glioblastoma Metabolism by Using Hyperpolarized [1-13C]Pyruvate Demonstrates Heterogeneity in Lactate Labeling: A Proof of Principle Study. Radiol. Imaging Cancer.

